# Smartphone- and internet-assisted self-management and adherence tools to manage Parkinson’s disease (SMART-PD): study protocol for a randomised controlled trial (v7; 15 August 2014)

**DOI:** 10.1186/1745-6215-15-374

**Published:** 2014-09-25

**Authors:** Rashmi Lakshminarayana, Duolao Wang, David Burn, K Ray Chaudhuri, Gemma Cummins, Clare Galtrey, Bruce Hellman, Suvankar Pal, Jon Stamford, Malcolm Steiger, Adrian Williams, The SMART-PD Investigators

**Affiliations:** uMotif Ltd, London, UK; Liverpool School of Tropical Medicine, Liverpool, UK; Regional Neurosciences Centre, Newcastle General Hospital, Newcastle upon Tyne, UK; Neurology/Movement Disorders, National Parkinson Foundation Centre of Excellence, King’s College Hospital, University Hospital Lewisham, Kings College and Institute of Psychiatry, London, UK; John van Geest Centre for Brain Repair, Department of Clinical Neurosciences, University of Cambridge, Cambridge, UK; St George’s Healthcare NHS Foundation Trust, London, UK; NHS Forth Valley, Anne Rowling Regenerative Neurology Clinic, University of Edinburgh, Edinburgh, UK; The Cure Parkinson’s Trust, London, UK; University of Liverpool and The Walton Centre NHS Foundation Trust, Liverpool, UK; University Hospitals Birmingham NHS Foundation Trust, Birmingham, UK

**Keywords:** Long-term conditions, m-health, Mobile application, Parkinson’s disease, Self-management

## Abstract

**Background:**

Nonadherence to treatment leads to suboptimal treatment outcomes and enormous costs to the economy. This is especially important in Parkinson’s disease (PD). The progressive nature of the degenerative process, the complex treatment regimens and the high rates of comorbid conditions make treatment adherence in PD a challenge. Clinicians have limited face-to-face consultation time with PD patients, making it difficult to comprehensively address non-adherence. The rapid growth of digital technologies provides an opportunity to improve adherence and the quality of decision-making during consultation. The aim of this randomised controlled trial (RCT) is to evaluate the impact of using a smartphone and web applications to promote patient self-management as a tool to increase treatment adherence and working with the data collected to enhance the quality of clinical consultation.

**Methods/Design:**

A 4-month multicentre RCT with 222 patients will be conducted to compare use of a smartphone- and internet-enabled Parkinson’s tracker smartphone app with treatment as usual for patients with PD and/or their carers. The study investigators will compare the two groups immediately after intervention. Seven centres across England (6) and Scotland (1) will be involved. The primary objective of this trial is to assess whether patients with PD who use the app show improved medication adherence compared to those receiving treatment as usual alone. The secondary objectives are to investigate whether patients who receive the app and those who receive treatment as usual differ in terms of quality of life, quality of clinical consultation, overall disease state and activities of daily living. We also aim to investigate the experience of those receiving the intervention by conducting qualitative interviews with a sample of participants and clinicians, which will be administered by independent researchers.

**Trial registration:**

ISRCTN45824264 (registered 5 November 2013)

**Electronic supplementary material:**

The online version of this article (doi:10.1186/1745-6215-15-374) contains supplementary material, which is available to authorized users.

## Background

### Parkinson’s disease

Parkinson’s disease (PD) is a degenerative movement disorder affecting 1% to 2% of the population over 60 years of age [1]. In one study on incidence rates, researchers reported that 0.5% of patients were diagnosed before the age of 40 years, 3.5% before the age of 50 years and more than 60% between the ages of 65 and 79 years [2]. The core motor features of PD comprise combinations of bradykinesia, resting tremor, rigidity, flexed posture, ‘freezing’ and loss of postural reflexes [3]. PD is a disabling condition with significant impact on quality of life (QoL) [4]. Notably, nonmotor symptoms of PD, such as dementia, sleep disturbances, depression and falls, may also have a significant negative impact on QoL [5]. Comorbidity is common in PD. Longitudinal studies have shown that people with PD have a three to six times higher risk of developing dementia than do people of the same age who do not have PD [6, 7]. PD is associated with significant burden of illness and cost to society. Increases in physician and drug costs and longer hospital admissions compared to age- and sex-matched controls have also been reported [8].

### Complexity of treatment

Managing both motor and non-motor symptoms of PD translates into complex treatment regimes. Treatment regimens are planned based on the stage of the illness- early or late- and on the type and mix of nonmotor symptoms [9]. The risk of side effects from treatment adds to the complexity of managing PD (Table 1). In early stages of the disease, patients usually take a single drug [9, 10] but in later stages of the disease more than half of the patients with PD take two to three drugs three to four times daily [11, 12].

**Table 1:** **Current available pharmacological treatment and side effects for early and late Parkinson’s disease** ^**a,b**^

Therapy	Risk of side effects	
	***Motor complications***	***Other side effects***
Adjuvant therapy for early PD		
Levodopa	↑**	↑***
Dopamine agonists	↓	↑
MAO-B inhibitors	↓	↑
β-adrenergic antagonists	Lack of evidence	
Amantadine	Lack of evidence	
Anticholinergics	Lack of evidence	
Modified-release levodopa	↑	↑
**Adjuvant therapy for late PD**		
Dopamine agonists	↓	↑
MAO-B inhibitors	↓	↑
COMT inhibitors	↓	↑
Amantadine	↓	↑
Apomorphine	↓	↑
Modified-release levodopa	↓	↑

^a^ *Adapted from* National Institute for Health and Care Excellence (NICE) [9]*.* ^b^COMT, Catechol-O-methyltransferase; MAO-B, Monoamine oxidase B; PD, Parkinson’s disease. ↑** Evidence of increased motor complications/other adverse events. ↓*** Evidence of reduced motor complications/other adverse events.

### Treatment nonadherence

Adherence to long-term therapies in the general population is around 50% in the developed world [13]. Nonadherence results in failed treatment outcomes and $100 billion spent each year worldwide on avoidable hospitalizations [14]. There are various predictors of medication nonadherence, including the following ones set forth by Osterberg and Blaschke [15]:

- Presence of psychological problems, particularly depression
- Presence of cognitive impairment
- Treatment of asymptomatic disease
- Inadequate follow-up or discharge planning
- Side effects of medication
- Patient’s lack of belief in the benefit of treatment
- Patient’s lack of insight into the illness
- Poor provider–patient relationship
- Presence of barriers to care
- Missed appointments
- Complexity of treatment
- Cost of medication, copayment or both

Adherence is inversely proportional to the frequency of dose, with patients taking medication on a schedule of four times daily achieving average adherence rates of about 50% [16]. Some clinicians propose that medication nonadherence should be conceptualised as a diagnosable and treatable medical condition [17]. The ability of physicians to recognize nonadherence is poor, and interventions aimed at improving adherence have had mixed results and are costly [15, 18].

Adherence to medication regimens is an essential prerequisite for symptom control in PD [19]. Rates of adherence to treatment in PD mimic those of other long-term conditions, with one research group reporting that 51.3% of subjects missed at least one dose per week and 20.5% of subjects missed three or more doses per week [20]. The authors of a systematic review of factors associated with nonadherence in PD patients reported six clinical factors (mood disorders, cognitive impairment, poor symptom control or reduced QoL, younger age or longer disease duration, and regime complexity or polypharmacy) and five demographic factors (lack of spouse or partner, low income, employment status, and gender) associated with nonadherence [19].

There are direct and indirect methods of measuring adherence. Direct methods include directly observed therapy, measurement of level of medicine or metabolite of medicine in blood, and measurement of biological markers in blood [17, 21]. Indirect methods include patient questionnaires, patient self-reports, pill counts, rates of prescription refills, assessment of patient’s clinical response, electronic medication monitors and patient diaries [17, 21]. Though certain methods are preferred in specific clinical or research settings, a combination of measures maximizes accuracy [22].

### Shared decision-making

Patients are increasingly taking active roles in decisions about major medical interventions, such as hip replacement surgery, and about routine decisions, such as medication initiation [23]. However, there is evidence on shared decision-making between patient and clinician that, though patients are well-advised to learn about the potential benefits and risks of relevant alternatives before a treatment decision is reached [24], this is not followed in most clinical settings due to clinician and patient factors [23, 25, 26]. In a large-scale survey of PD patients in Sweden, researchers found that doctors provided only a small proportion of patients with advanced therapy information, despite patients’ interest [27].

### Digital health care

The use of digital technology in daily life has become ubiquitous. As of 2012, 76% of the UK population had access to broadband connections and 92% of adults personally owned or used mobile phones, of which 60% were smartphones (that is, mobile phones with features of a handheld computer, such as internet access, data storage, email capability and voice and video recording) [28, 29]. The authors of a recent systematic review on the use of mobile phones in health care reported that voice and text messaging services are used to improve health outcomes (medication compliance, asthma symptoms, haemoglobin A1C control, stress levels, smoking quit rates, and self-efficacy) and processes of care (fewer missed appointments, quicker diagnosis and treatment, and improved teaching and training) [30]. Evidence on the use of smartphones to improve clinical outcomes is also gathering momentum [31]. The push to use digital technologies in the UK National Health Service (NHS) to improve clinical outcomes has increased in recent years, with the Whole Systems Demonstrator study investigators reporting that telehealth services substantially reduce mortality, the need for hospital admissions, the number of bed days spent in hospital and the time spent in the accident and emergency department [32].

Smartphones have been used in managing PD to assess pervasive movement analysis remotely [33] and for predicting the risk of falls [34]. The SMART-PD trial was preceded by a 2 –month pilot trial carried out by uMotif Ltd, Birkbeck University of London and The Cure Parkinson’s Trust. Here researchers evaluated the impact of a patient-led tracker mobile application (an ‘app’) to improve medication regime adherence among PD patients from December 2012 to March 2013 [Lakshminarayana R, Hellman B, Addyman C, Stamford J Self-management in long-term conditions using smartphones: A pilot randomised trial in Parkinson’s disease (submitted)]. A total of 36 patients with PD took part in a 55-day pilot study and were randomised into two groups. The limited-app group (*n* = 19) received an app which had tools for daily self-tracking on ten measures of symptom severity, general well-being and health behaviours, along with a daily diary. The full-app group (*n* = 17) received an app which had the same tools as the limited-app group with the addition of medication reminders and two games to assess cognition. Participants used the app for 55 days and entered data on at least 70% of those days.

### Study rationale

Despite evidence highlighting nonadherence to medication regimes among patients with PD and a lack of evidence on shared decision-making, there is little evidence on interventions that improve adherence to PD treatment. Following up on the findings of the 2-month pilot trial study on patient-led use of a tracker app [35], in this follow-up study we aim to evaluate the impact of using a Parkinson’s tracker app (PTA) along with treatment as usual (TAU) on medication adherence in a larger number of patients across multiple trial sites.

We hypothesise that encouraging patients to track their symptoms and medication intake regularly may result in increased medication adherence. Furthermore, we postulate that using data collected by the patient may lead to better symptom control, an improved QoL and a higher quality of clinical consultation.

### Trial objectives

#### Primary objectives

Our primary objective is to investigate whether patients with PD who use the PTA in addition to TAU show improved medication adherence.

#### Secondary objectives

A secondary objective is to investigate whether patients who receive the PTA and those who receive TAU differ in terms of QoL, quality of clinical consultation, overall disease state, activities of daily living (ADL), beliefs about medication and generic health-related QoL. We also aim to investigate the experiences of those receiving the intervention by gathering information from qualitative interviews administered by independent researchers.

## Methods/Design

A single-blind, multicentre RCT will be conducted to compare use of smartphone- and internet-enabled PTA with TAU among patients with PD and/or their carers across seven centres in England and Scotland. The trial has received ethical approval from the National Research Ethics Service London–Westminster Research Ethics Committee (13/LO/1783).

### Trial participants

Patients diagnosed with PD attending neurology outpatient (OP) appointments or research clinic appointments at participating sites will be enrolled into the trial over a 16-week period. We have adapted the inclusion and exclusion criteria from a similar study on treatment adherence in PD patients [35].

To be eligible for the trial, patients must meet the following criteria: (1) older than 21 years of age; (2) diagnosed with probable, idiopathic PD; (3) prescribed one or more antiparkinsonian medications; (4) English-speaking and literate (that is, can read, write and speak in English and compute and solve problems at levels of proficiency necessary to function daily at a job and/or in ADL); (5) have access to a smartphone and/or tablet or internet on a daily basis at home; (6) be on a stable medication regime (that is, not altered within the previous month and not expected to change during the period of the trial (16 weeks), with alteration of doses of existing medications considered to be a stable medication regime); and (7) without a diagnosis of dementia or significant cognitive impairment (as recorded in patient case file). The clinical team will judge whether the patient has the cognitive capacity required to participate fully in the trial (that is, read patient information, complete self-report questionnaires and sign informed consent form).

The following are the exclusion criteria: (1) suspected parkinsonism due to causes other than idiopathic PD, (2) current or previous treatment with antiparkinsonian medications (anticholinergics) for side effects of prolonged neuroleptic treatment, (3) diagnosis of dementia or significant cognitive impairment (as recorded in patient case file), (4) current or previous diagnosis of mental illness associated with psychosis (schizophrenia, severe depression with psychosis, bipolar affective disorder) (as recorded in patient file), and (5) detrimental illness with a short life expectancy.

Informed consent will be obtained from every trial participant.

### Outcome measures

The primary outcome measure is adherence to treatment as determined by Morisky Medication Adherence Scale (MMAS-8) score at 16 weeks [36, 37]. The response categories are ‘yes’ or ‘no’ for each item with a dichotomous response and a 5-point Likert response for the last item. A ‘yes’ response is scored as 1 and a ‘no’ has no score. The primary endpoint will be the total MMAS-8 score at 16 weeks. We are collecting data from a range of secondary outcome measures, including the following:

- Parkinson’s Disease Questionnaire (PDQ-39) to measure QoL [38]
- Quality of consultation for PD patients (from the *Patient-Centered Questionnaire for PD*[39]
- Non-Motor Symptoms Scale [40]
- Hospital Anxiety and Depression Scale [41]
- Beliefs about Medication Questionnaire [42]

In addition to the measures listed above, we will collect demographic data at baseline, such as participant age, duration and severity of PD (Unified Parkinson’s Disease Rating Scale score if recorded in clinical file), medication profile (types of medication, dosage level and frequency), socioeconomic status, ethnicity, comorbidities, level of education and whether medication is self-administered or given by a spouse or carer. We also will collect patients’ self-reported ratings and game scores within the application.

### Randomisation and masking

Eligible patients for whom consent or assent is provided will be allocated in a 1:1 ratio to the two arms of the study according to a computer-generated random sequence stratified by centre and using blocks of variable size. The allocation sequence will be generated by the trial statistician and will not be available to any member of the research team until databases have been completed and locked. In view of the established safety record of study interventions, there will be no provision for emergency unblinding of participants, and copies of the allocation sequence will not be held at the recruiting centres.

### Recruitment procedures

Patients who are potentially eligible based on inclusion criteria (except inclusion criterion 4) will be identified by the clinical team across the seven trial sites from the clinic’s list 6 weeks prior to upcoming outpatient appointments. An information pack containing a patient invitation letter, a Participant Information Sheet and a consent form will be sent to potential participants 3 to 4 weeks prior to the next OP clinic appointment or separately with an OP clinical appointment date by post and/or by email, along with a request to reply within 1 week. Reminders will be sent by post and/or email sent 1 to 2 weeks before an OP appointment to those who fail to respond to email. At the OP appointment, the clinician will (1) recheck whether the participant has daily access to a smartphone or tablet or an internet connection and (2) assess whether a change in medication regime is needed. If the patient does not have such a connection and/or needs a change in medication regime, he or she will not be enrolled into the trial. However, these participants will be given free access to the app once the trial is completed. Patients sign a hard copy of the consent and complete the questionnaires at the clinic if it is possible to give them internet access at the clinic or if they can complete the questionnaires at home within 1 week after the OP appointment. If participants withdraw from the trial after giving consent, they will continue to receive the treatment they were receiving prior to the start of the trial (that is, TAU). Data collected up to the point of withdrawal will be retained for final analysis. Following consent, patients are randomised to either the intervention or the control group. Randomisation is carried out with sealed randomisation envelope for each participant generated by the trial statistician. The clinician runs through the details of the allocation—intervention or control—with the patient and gives him or her the date for follow-up. Recruitment will be a rolling programme over the course of 16 weeks until recruitment targets are achieved. Participants in the intervention group will get a call from their clinicians 2 weeks postrandomisation to check if they have any difficulties with the PTA. All participants get a call from their clinician 1 to 2 weeks prior to the follow-up appointment to remind them of their appointment. Participants complete the questionnaires within 1 week after their appointment.

**Figure 1:**
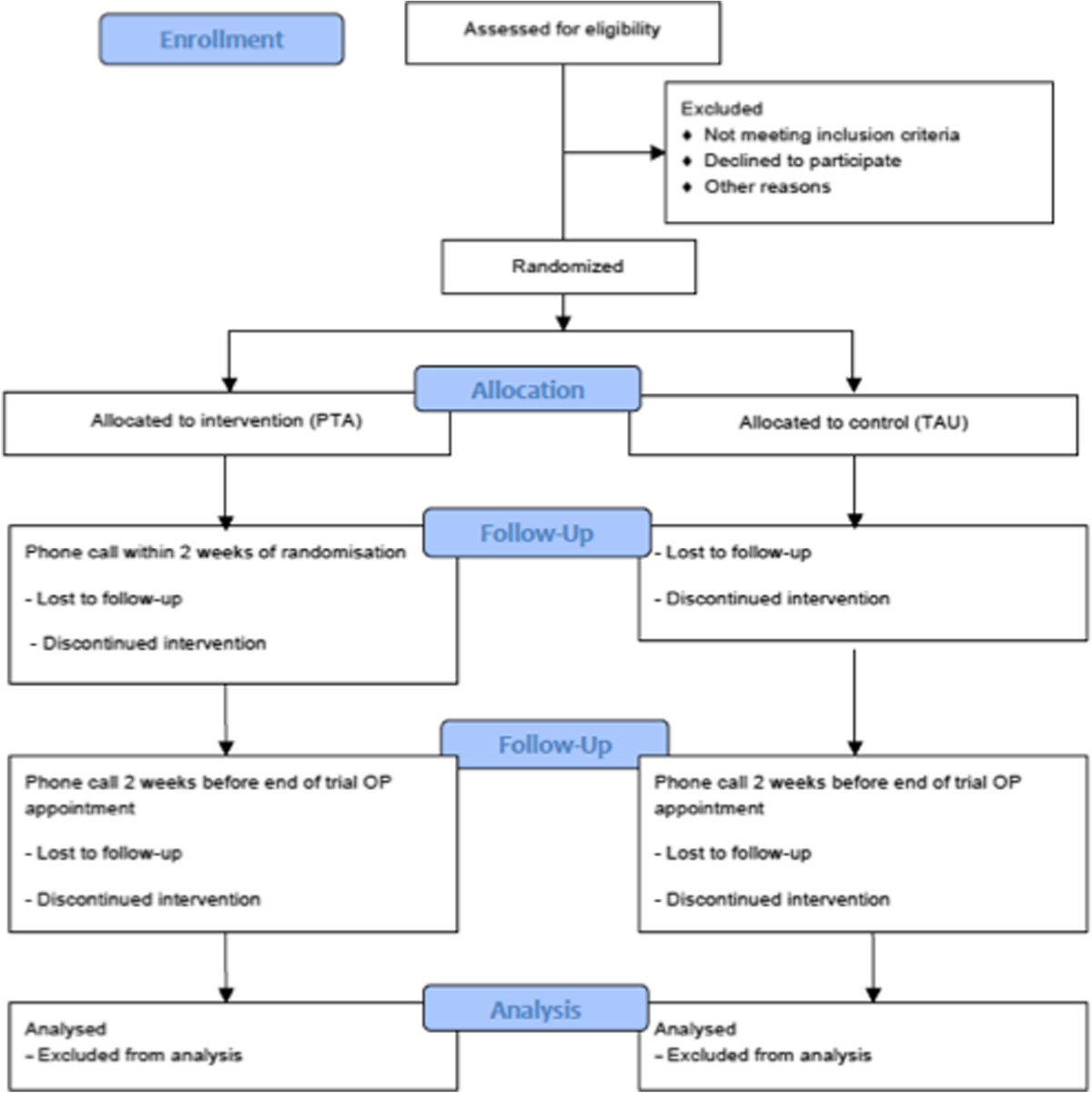
**CONSORT flowchart for the SMART-PD trial.** CONSORT, Consolidated Standards of Reporting Trials; OP, Outpatient; PTA, Parkinson’s tracker app; SMART-PD, Smartphone- and internet-assisted self-management and adherence tools to manage Parkinson’s disease; TAU, Treatment as usual.

The trial is being carried out in adherence to the Consolidated Standards of Reporting Trials (CONSORT) Statement [43] (Figure 1), and the protocol adheres to the guidelines for clinical trial protocols set forth in the SPIRIT 2013 Statement (Standard Protocol Items: Recommendations for Interventional Trials) Additional file 1: Table S1, [44].

### Sample size

The sample size for the smartphone- and internet-assisted self-management and adherence tools to manage Parkinson’s disease (SMART-PD) trial has been determined in a pragmatic manner based on available funding to demonstrate both the effectiveness and the ease of implementation of the intervention. The sample size was calculated along two dimensions: the primary outcome (that is, MMAS-8 score) and the secondary outcome (QoL). The main sample size calculation was based on the primary endpoint. To detect a 1-point improvement on the MMAS-8 with a standard deviation (SD) of 2.5 and 80% power at the 5% significance level would require 200 subjects (100 in each group, 1:1 allocation). To allow for 10% loss due to dropouts and those lost to follow-up, we would need to recruit 222 subjects (111 in each group, 1:1 allocation). The study is also powered to detect a 6-unit difference in the secondary outcome, QoL (PDQ-39). With a SD of 9 and 80% power at the 5% significance level, 74 subjects would be required (37 in each group, 1:1 allocation). To allow for 10% loss due to dropouts and those lost to follow-up, we would need to recruit 82 subjects (41 in each group, 1:1 allocation). Therefore, 222 participants will be recruited.

### Intervention group

#### Parkinson’s tracker app

**Figure 2:**
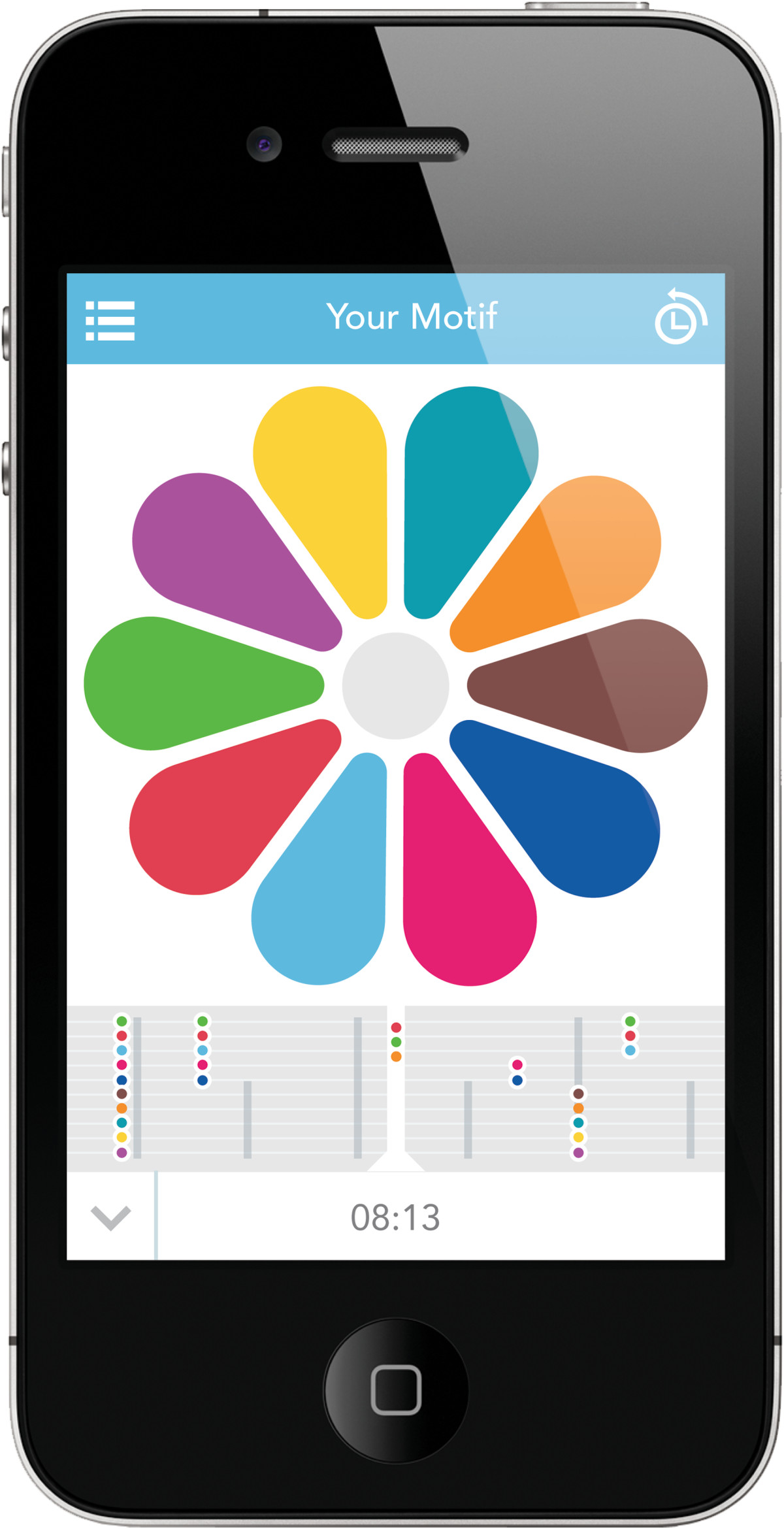
**Screenshot of the self-tracking interface.**
- A reminder system for patients to receive alerts and track medication intake
- An option to generate a compiled report of data entered by the patient over the trial period that will serve as an aid for shared decision-making during their follow-up outpatient appointment
- Games to track physical responsiveness (finger-tapping task) (Figure 3) and cognition (number-size Stroop test) (Figure 4)

Participants allocated to receive the intervention will receive instructions to download the PTA to their Android or iPhone smartphones or tablet devices or to access it via a website portal within 1 day after they have attended their outpatient appointment. The app consists primarily of the following features:

•Using a sliding petal interface (Figure 2), participants will adjust their daily scores on eight to ten self-monitoring measures on a 5-point scale: water, sleep, exercise, 5 A DAY healthy diet, mood and energy, medication, movement, suppleness and control. Participants who have difficulty manipulating the flower icon due to their motor symptoms will have the option of using an accessibility mode with a zoom function to magnify the screen. Participants will be able to review their scores and compare ‘petals’ against each other.

**Figure 3:**
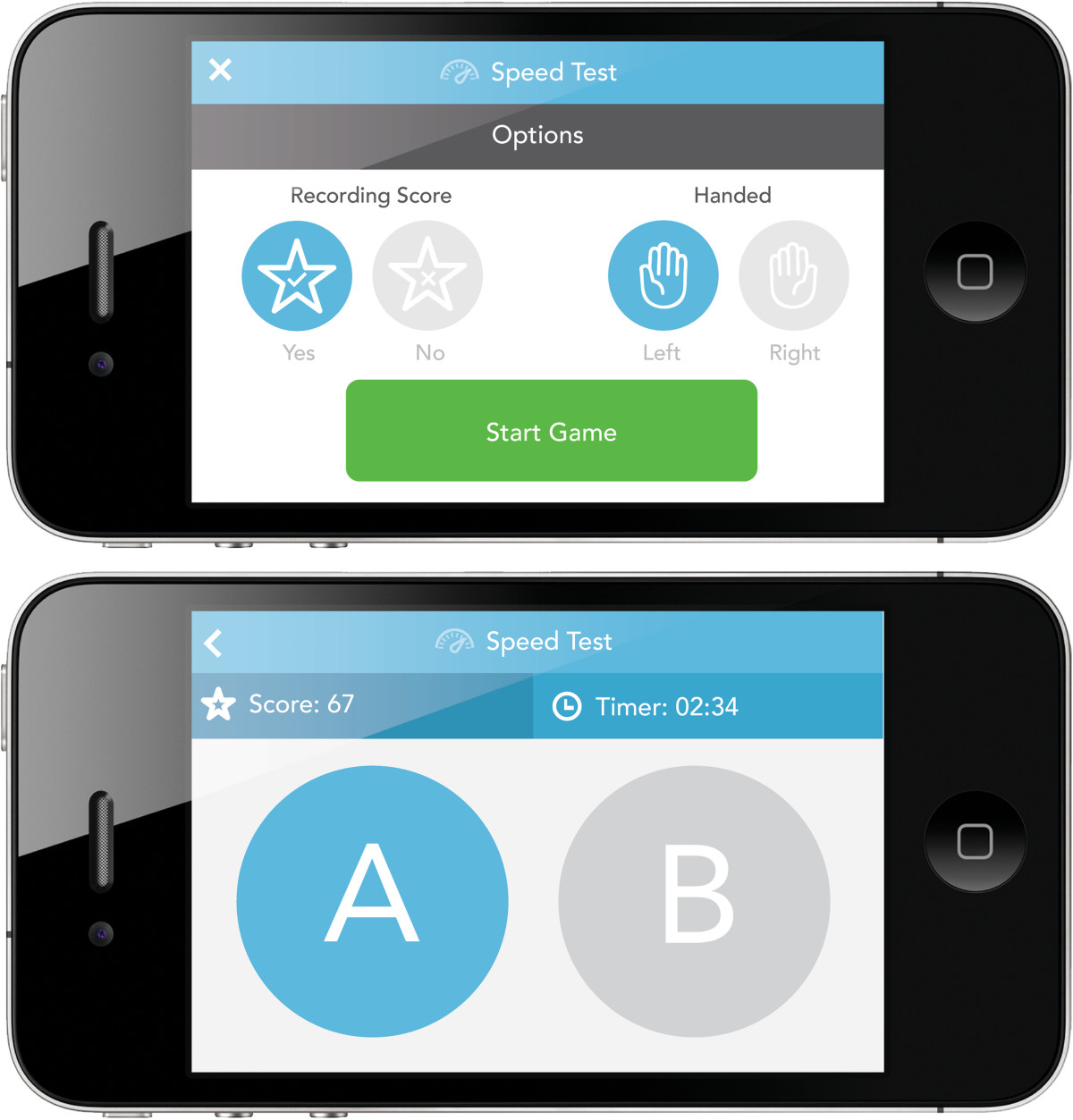
**Screenshots of the finger-tapping test.**

**Figure 4:**
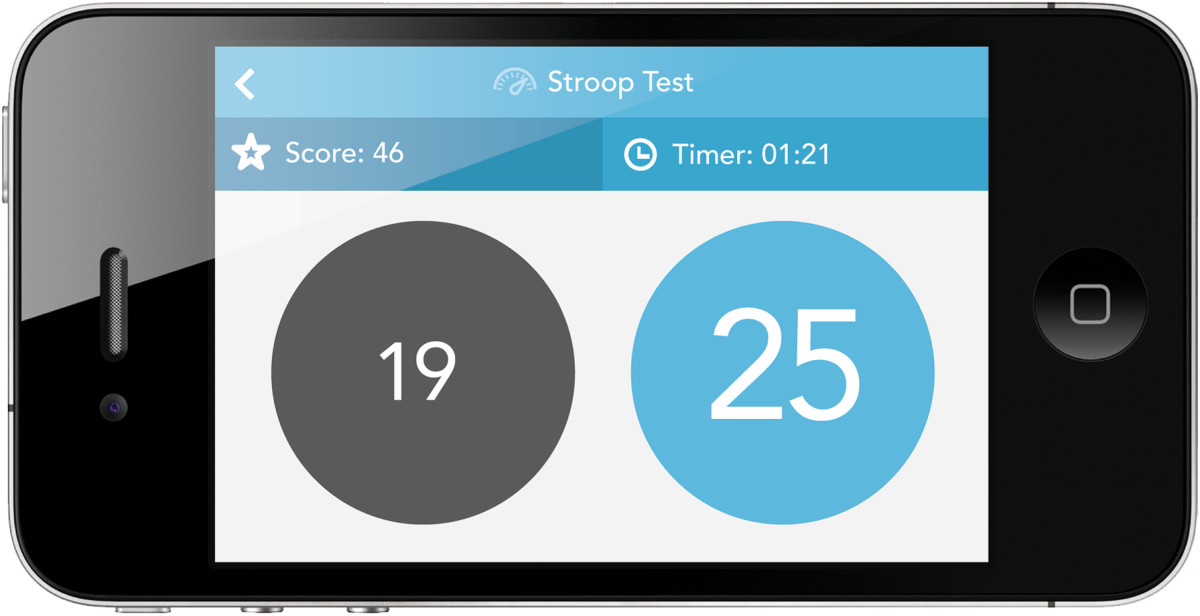
**Screenshots of the number-size Stroop test.**

##### Finger-tapping test

In the finger-tapping test, participants have to tap the screen of the smartphone as many times as they can in a 20-second period, alternating between two circular targets. The game displays the participant’s cumulative score. The app also records variations in responses.

##### Number-size Stroop test

In the number-size Stroop test, the game displays two circular targets, each containing a number in the range 1 to 99 in various font sizes. Participants have to tap the *numerically* larger value and visual size information. Participants get points for correct answers when they select the numerically larger number. The game lasts 20 seconds and displays the cumulative score. The app also records reaction times and variability in responses.

#### Tracking progress

**Figure 5:**
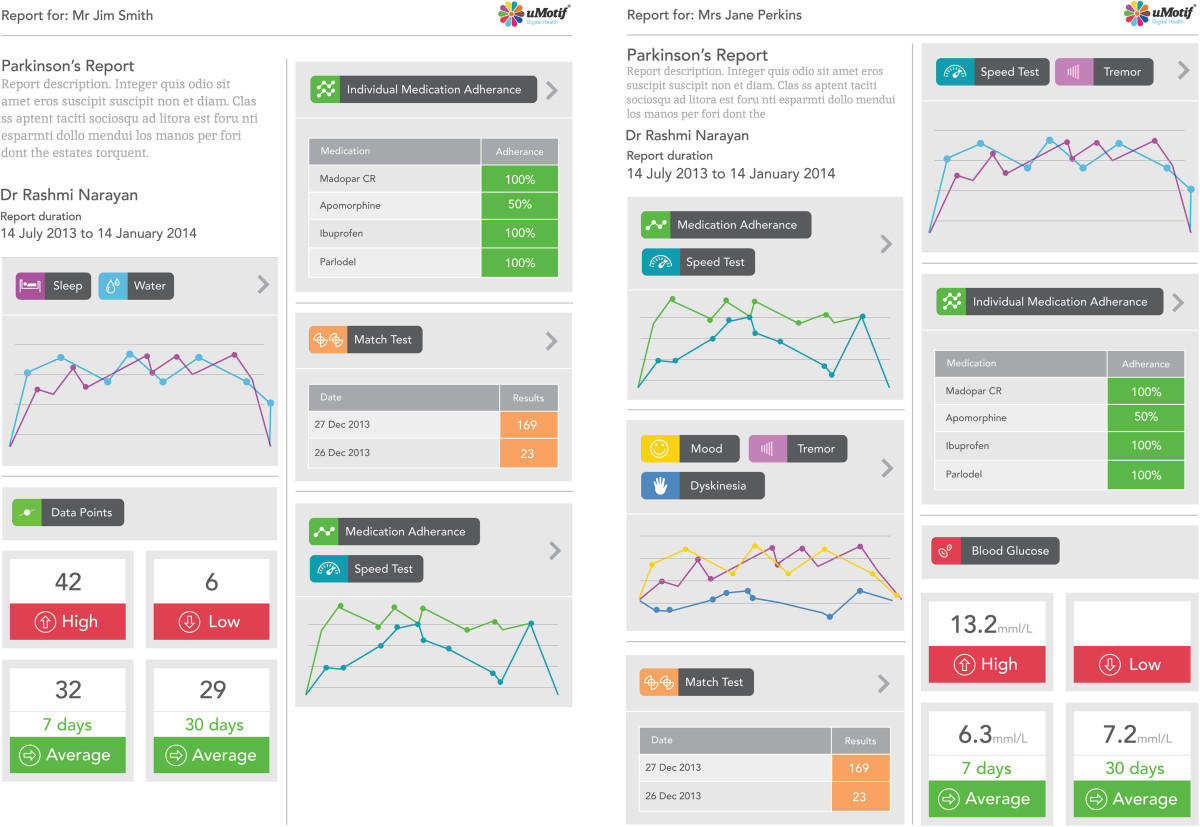
**Screenshot of a sample report generated by the Parkinson’s tracker app.**

Clinicians will have access to a dedicated web portal where they can see participants’ progress over the trial period. Participants will also have access to a dedicated web portal. However, neither group will be able make changes to any data. At the follow-up appointment, participants in the PTA group will be able to generate a report compiled from the data they have entered over the trial period to share with their clinicians (see Figure 5).

### Treatment-as-usual group

They will have their regular OP clinical assessments, including symptom review followed by a medication review, at the start of the trial and at the end of 16 weeks. We do not expect any adverse events to occur during the trial.

### Statistical analysis

The trial statistician will be blinded to the allocation of trial participants. In the main analysis, the effect of the interventions on the primary and secondary outcomes will be assessed. The generalised linear model (GLM) will be employed for analysis of the primary endpoint (MMAS-8 at 16 weeks). The GLM model will have treatment as the fixed effect and baseline measurement of the primary endpoint as the covariate. For GLM model analysis, normal distributions will be used in the GLM, and the mean difference in MMAS-8 with its 95% confidence interval will be derived and reported. Model assumptions about residuals in regression analysis will be checked by inspection of residuals versus a fitted values plot. In addition, adjusted analysis and subgroup analysis with prespecified covariates will be performed on the primary endpoint analysis.

For the secondary outcomes (QoL, depression, anxiety, nonmotor symptoms, and degrees of depression and anxiety), the analyses will be performed in an analogous fashion within the framework of GLM. In addition, summary statistics will be generated for the primary and secondary endpoints with number (%) expressed for binary outcomes and number, mean and SD used for continuous outcomes.

Analysis of the primary and secondary outcomes will be carried out in adherence to the intention-to-treat principle (that is, the participants will remain in the group to which they were randomised and not analysed according to the interventions actually received). In addition, supplemental per-protocol analyses will be performed. Detailed results of statistical analysis will be described in the statistical analysis plan, which will be finalised before lockup of the database. The SAS 9.2 statistical software package (SAS Institute, Cary, NC, USA) will be used for all the data analyses.

### Exploratory analyses

Self-reported scores and game data from the PTA provide an opportunity to perform exploratory analysis of patterns of day-to-day variation and correlation of Parkinson’s symptoms with medication intake and other health behaviours. Previous researchers have found that daily self-tracking and data from the finger-tapping test give a detailed and sometimes conflicting picture of a patient’s condition compared to retrospective self-assessment [45]. Data recorded daily within the PTA will be used to examine correlations between various items collected via the PTA at individual and group levels. These scores will also be used in regression analyses against the primary and secondary outcome measures.

### Qualitative evaluation

Semistructured interviews will be undertaken by an independent agency with a purposively selected subsample of participants (*n* ≤ 5) to explore the process and experience of using the PTA. We will interview only participants who consent to be interviewed. All participants who give us consent will be pooled, and those who will be interviewed will be selected at random to minimize selection bias. Interviews will be 45 minutes in duration. The aims are to complement the quantitative findings by obtaining insights into the experience of using the PTA, consider which elements of the PTA are most helpful, explore perceptions of how the PTA has influenced medication taking and explore how the PTA could be improved. Exploratory correlational analysis will be performed on the self-report and game score data collected by using the PTA.

### Ethical considerations

We do not anticipate any serious risk to trial participants, as we assume that patients will take their medications as prescribed by their clinicians. However, there is a possibility that increased medication adherence in PD may lead to a greater incidence of medication induced side effects, such as dyskinesia, postural hypotension, confusion, nausea and impulsive and/or compulsive behaviour disorders resulting from dopaminergic therapy for PD. We will alert patients about these potential side effects in the Patient Information Sheet and advise them to contact their clinical team, if necessary, as part of routine clinical practice.

### Trial monitoring

We will assess the impact of using self-management tools delivered via smartphones and the web, along with routine treatment, for 16 weeks. The trial protocol has minimal to no risk of worsening PD progression. These factors led us to form a monitoring group rather than a Data Monitoring Committee. Monitoring of the trial will be performed by the Trial Management Group (TMG), which will consist of lead investigators from each site and the Chief Investigator (CI) from uMotif. The CI will call and/or email each trial site every 2 weeks to check if there have been any issues with software and to follow up on recruitment rates. The CI will send a monthly update to each of the trial sites and to the NHS research and development lead. The trial advisory board will include members of the TMG and representatives from The Cure Parkinson’s Trust UK and Parkinson’s UK. It will be chaired by Dr Jon Stamford from The Cure Parkinson’s Trust. We have scheduled three TMG meetings—one prior to the start of the trial, one midway through and one at the end of the trial. Any issues related to the software, participants or the trial, including protocol amendments, will be discussed and resolved by the TMG at the meeting scheduled for midway through the trial.

### Trial administration

Administration of the trial reflects the allocation of responsibilities set out in the Research Governance Framework [46]. The CI will be in charge of conducting the trial according to the agreed protocol and in accordance with legal requirements, guidance and accepted standards of good practice; will arrange to make findings and data accessible following expert review; and inform participants of the research results. Lead investigators at the trial sites will conduct the trial according to the agreed protocol and in accordance with legal requirements, guidance and accepted standards of good practice; will ensure participants’ welfare while in the study; and will arrange to make findings and data accessible following expert review. The trial sponsor is responsible for establishing and keeping in place arrangements to initiate, manage and fund the study.

### Data management

Data will be stored securely on hardened servers and backed up. To maintain data protection, the sponsor has notified the Information Commissioner’s Office that it is a data controller and processor. No data will be shared with anyone or any entity other than the trial team. Data sets will be anonymised before transfer to the outcomes assessor (that is, the trial statistician) at the end of the trial. The trial statistician and sponsor will have access to the final data set.

### Publication policy

#### Reporting and dissemination

Prior to submission or application for presentation, all manuscripts, posters, oral presentations and other reports of the outcomes of this research effort will be approved uMotif. All publications will include a formal acknowledgement that the trial was designed by uMotif with feedback from trial sites, and the Liverpool School of Tropical Medicine and that it was or is being carried out by uMotif and all trial sites. We will also acknowledge that the trial was supported by The Cure Parkinson’s Trust and Parkinson’s UK and that the study was commissioned by NHS Midlands and East and funded by the UK Department of Health.

#### Authorship

The authorship of manuscripts, posters, oral presentations and any other reports of the results of this study will be guided by the criteria for authorship formulated by the International Committee of Medical Journal Editors as published in its *Recommendations for the Conduct, Reporting, Editing, and Publication of Scholarly Work in Medical Journals*[47]. According to these requirements, the authors should meet the following criteria: (1) Each author should have participated sufficiently in the work to take public responsibility for the content; and (2) authorship credit should be based only on substantial contributions (a) to the conception and design or analysis and interpretation of data; (b) to the drafting of the manuscript or to revising it critically for important intellectual content; and (c) by giving final approval of the version to be published. Conditions 2a), 2b) and 2c must be met. A plain English summary of the findings will be sent to all trial participants.

## Discussion

There is a paucity of evidence on interventions to enhance treatment adherence in PD. Unlike previous studies in which researchers investigated adherence to therapy programmes, SMART-PD uses resources that patients and their carers and/or partners have (i.e. a smartphone or tablet device). By ensuring that the intervention is introduced in routine clinical practice with minimal need for additional resources, our aim in the SMART-PD trial is to demonstrate that adherence to treatment and quality of clinical consultation can be improved in a cost-effective manner. The subjective nature of the self-report instruments used in the trial is acknowledged. Patients may over- or underreport their true health status, depending on the trial arm to which they are assigned. To reduce this bias, baseline primary outcome measures will be completed within a maximum of 2 weeks of randomisation. The nature of the SMART-PD intervention does not allow masking of study participants blinded to their group allocation. Therefore, secondary outcomes at baseline (completed postrandomisation) and all follow-up study outcomes will not be blinded. The SMART-PD intervention is aimed at PD patients who have smartphones and/or tablet devices or internet access, thereby omitting those who do not have such access. To reduce this selection bias, we are including those patients whose carers and/or partners have a smartphone. However, with rapid penetration of smartphones in the UK, we expect this limitation to decrease rapidly. If effective, the trial results will demonstrate improved outcomes with the use of innovative smartphone technology for patients with long-term conditions and the clinicians who treat them.

## Trial status

Recruitment began 12 August 2014. It is anticipated that study recruitment will be completed by 31 October 2014 and that the trial will conclude by 30 April 2015.

## Electronic supplementary material

Additional file 1: Table S1: SPIRIT 2013 Checklist: Recommended items to address in a clinical trial protocol and related documents. (DOCX 39 KB)

Below are the links to the authors’ original submitted files for images.

Authors’ original file for figure 1

Authors’ original file for figure 2

Authors’ original file for figure 3

Authors’ original file for figure 4

Authors’ original file for figure 5

## Abbreviations

ADL: Activities of daily living
CI: Chief Investigator
CONSORT: Consolidated Standards of Reporting Trials
GLM: Generalised linear model
MMAS-8: 8-item Morisky Medication Adherence Scale
OP: Outpatient
PD: Parkinson’s disease
PDQ-39: 39-item Parkinson’s Disease Questionnaire
PTA: Parkinson’s tracker app
QoL: Quality of life
RCT: Randomised controlled trial
TAU: Treatment as usual
TMG: Trial Management Group.
