# Supplementary figures and images for: Smartphone- and internet-assisted self-management and adherence tools to manage Parkinson’s disease (SMART-PD): study protocol for a randomised controlled trial (v7; 15 August 2014)
Source: Trials. 2014 Sep 25;15:374. doi: 10.1186/1745-6215-15-374 (PMC4283131; doi:10.1186/1745-6215-15-374)

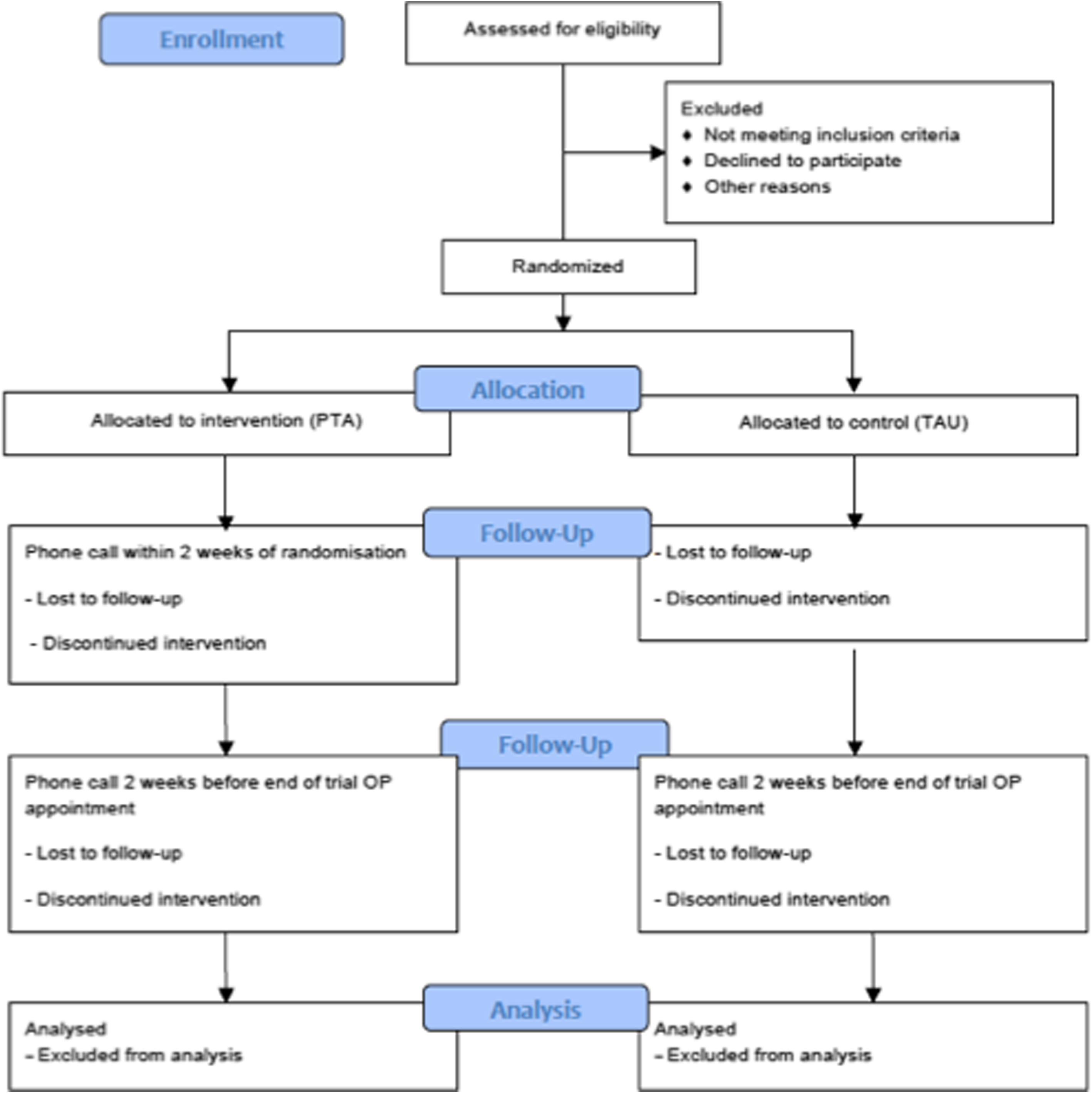

Supplement: Supplementary file 2 — Authors’ original file for figure 1 [file 13063_2014_2341_MOESM2_ESM.tiff]

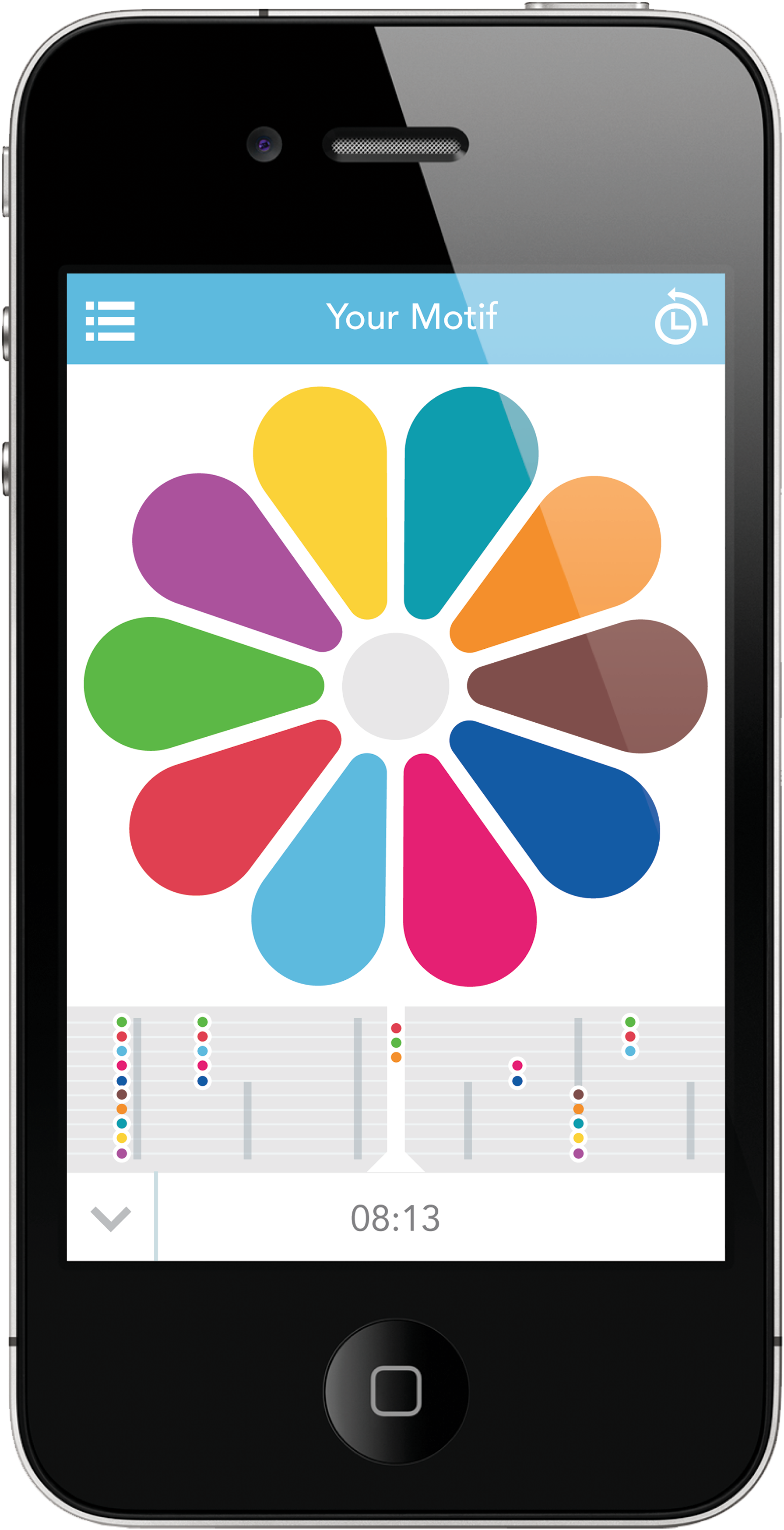

Supplement: Supplementary file 3 — Authors’ original file for figure 2 [file 13063_2014_2341_MOESM3_ESM.tiff]

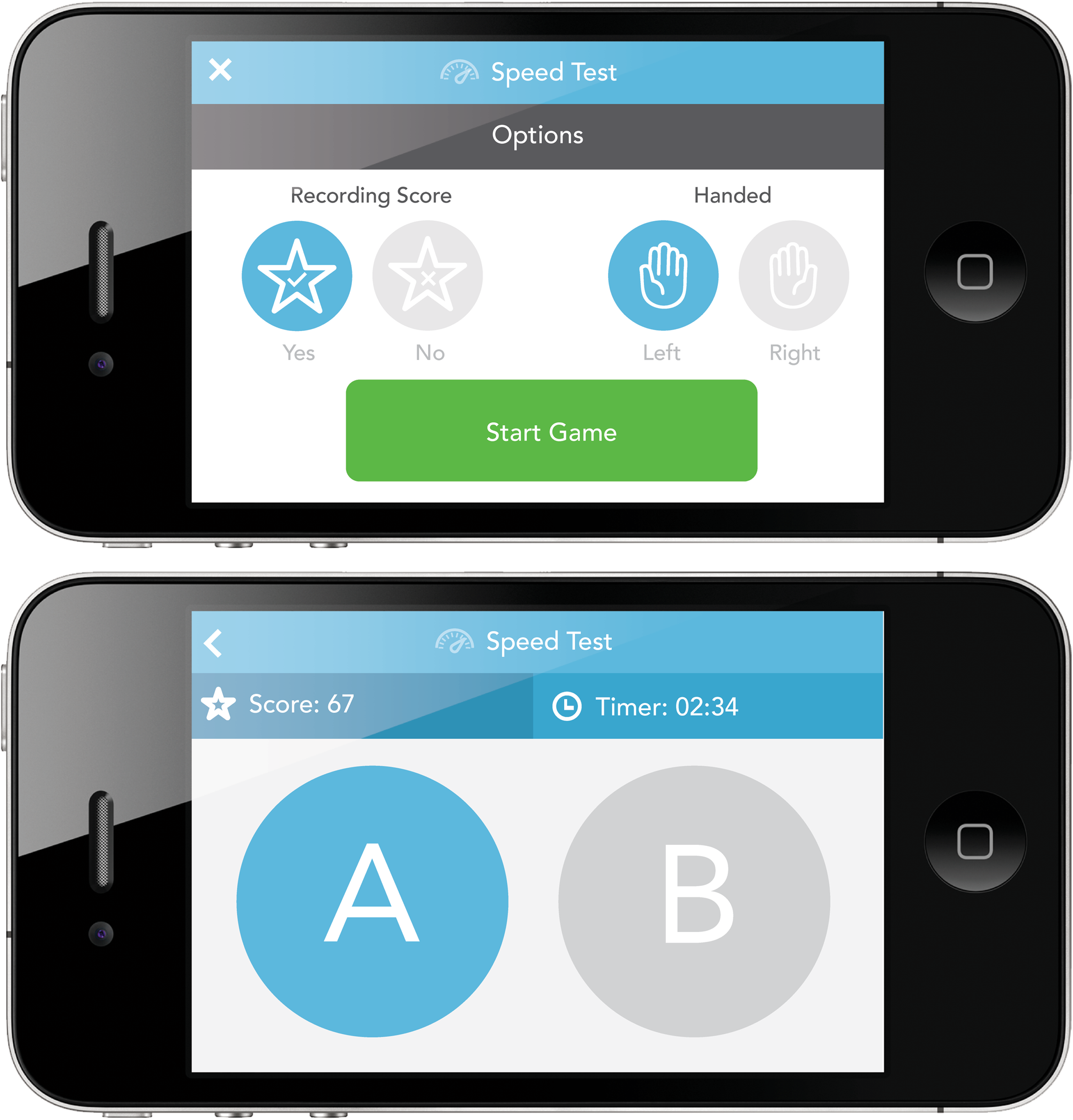

Supplement: Supplementary file 4 — Authors’ original file for figure 3 [file 13063_2014_2341_MOESM4_ESM.tiff]

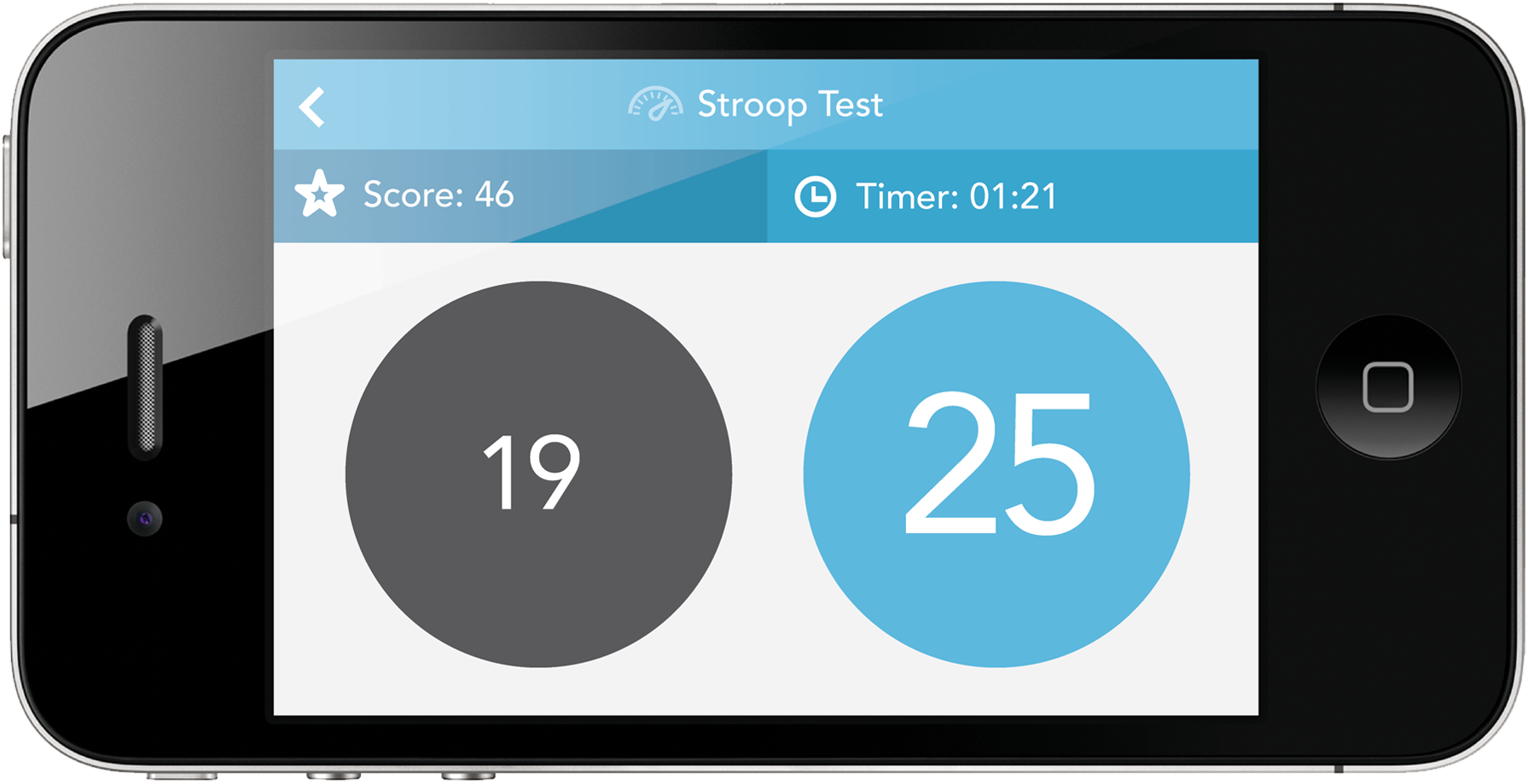

Supplement: Supplementary file 5 — Authors’ original file for figure 4 [file 13063_2014_2341_MOESM5_ESM.tiff]

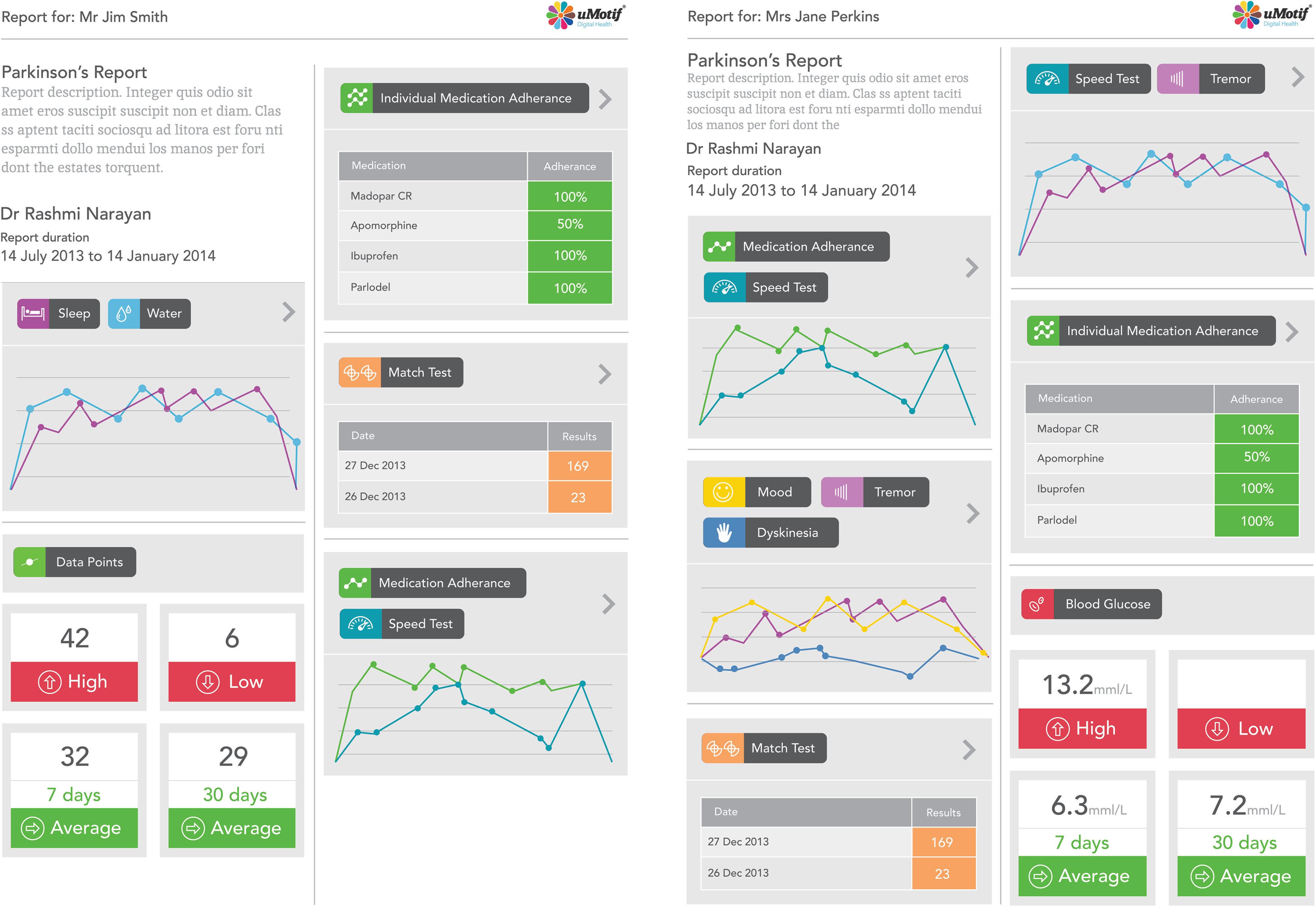

Supplement: Supplementary file 6 — Authors’ original file for figure 5 [file 13063_2014_2341_MOESM6_ESM.tiff]
